# Bridging early development gaps in rural Egypt: a community-based approach to equitable childhood care

**DOI:** 10.1186/s12939-025-02728-4

**Published:** 2025-12-18

**Authors:** Ammal M. Metwally, Ebtissam M. Salah El-Din, Marwa W. Abouelnaga, Maysa S. Nassar, Manal A. Shehata, Doaa E. Ahmed, Ghada A. Elshaarawy, Nihad A. Ibrahim, Ayman F. Armaneous, Mona A. Elabd

**Affiliations:** 1 Community Medicine Research Department/ Medical Research and Clinical Studies Institute, National Research Centre (Affiliation ID: 60014618), Dokki, Cairo Egypt; 2 Child Health Department/ Medical Research and Clinical Studies Institute, National Research Centre (Affiliation ID: 60014618), Dokki, Cairo Egypt

**Keywords:** Early childhood development, Health equity, Community-Based intervention, Rural health, Family care indicator, Developmental screening, Egypt

## Abstract

**Background:**

Early childhood development (ECD) is a critical foundation for health, learning, and social well-being. In Egypt, many children suffer from developmental delays, particularly in rural areas where early nurturing care is neglected.

**Aim:**

This study evaluated the effectiveness of a community-based intervention, based on the framework of WHO/UNICEF Care for Early Child Development, in improving caregiving practices and developmental outcomes among children under six years in a rural Egyptian district.

**Methods:**

A quasi-experimental longitudinal study with a post-test-only control group was implemented in one intervention and one control village. The intervention involved structured caregiver education, skill-building sessions, and continuous home-based support delivered by trained community health workers. Family Care Indicators (FCIs) from the Multiple Indicator Cluster Surveys and child development assessments using the Denver II Screening Tool were applied to evaluate progress. Statistical analyses were conducted using SPSS v24. Descriptive comparisons used Chi-square and paired t-tests. Multivariable logistic regression analyses were performed to control for potential confounders and to identify independent predictors of positive family care and developmental outcomes. Adjusted odds ratios (AORs) with 95% confidence intervals (CIs) were calculated, with significance set at *p* ≤ 0.05.

**Results:**

Adjusted regression analyses showed significant associations between intervention participation and improved outcomes across all domains. Families in the intervention village had higher odds of **Availability of ≥ 3 children’s books** (AOR = 3.41, 95% CI 1.87–6.24), and engaging in ≥ 4 caregiver–child interactive activities (AOR = 3.22, 95% CI 1.97–5.25). Children carried more odds to attend preschool (AOR = 3.76, 95% CI 2.01–7.02) and demonstrate normal fine-motor (AOR = 3.18, 95% CI 1.92–5.26), language (AOR = 2.64, 95% CI 1.63–4.27), gross-motor (AOR = 2.93, 95% CI 1.75–4.91), and personal–social development (AOR = 3.83, 95% CI 2.07–7.09), (all *p* < 0.001).

**Conclusion:**

Participation in the community-based program was positively associated with improved nurturing-care practices and developmental performance after controlling for key sociodemographic factors. The findings suggest that integrating similar caregiver training and early stimulation programs into national child health strategies may help promote equitable early childhood development.

**Supplementary Information:**

The online version contains supplementary material available at 10.1186/s12939-025-02728-4.

## Introduction

Child development during the first 1,000 days of life plays a foundational role in shaping lifelong health, cognitive capacity, and socioemotional outcomes [1, 2]. This early period of brain development is widely recognized as a unique and cost-effective window for interventions that can enhance future wellbeing and productivity [3].

The developmental process spans multiple domains; physical, cognitive, language, and socio-emotional which are deeply interconnected. For example, exploration enhances cognitive skills, while verbal communication fosters emotional bonding [4]. A variety of genetic, nutritional, and environmental influences affect these trajectories.

According to the Nurturing Care Framework for Early Childhood Development, approximately 40% of children under five in low- and middle-income countries lack adequate nurturing care; defined by deficits in health, nutrition, responsive caregiving, security, and early learning placing them at heightened risk for suboptimal development [5]. Furthermore, Draper et al., 2024 reported that just 62 million children (25.4%) in low and middle-income countries (LMICs) currently receive sufficient nurturing care throughout the age range from two to five years, putting 181.9 million children at risk for problems that could compromise their healthy development [6].

Socio-environmental contexts; including parenting practices and home environments are critical determinants of early childhood development [3, 7]. Secure, responsive caregiving fosters physical and intellectual growth [8]. In particular, parental engagement in stimulating activities such as reading, singing, storytelling, and playing is associated with enhanced cognitive and socioemotional outcomes [9, 10]. Conversely, early exposure to neglectful or harsh environments predicts language delays and behavioral issues [11]. Broader cultural and community norms also influence childrearing practices and the prioritization of education and social support [12].

In Egypt, multiple barriers hinder early childhood development (ECD): poor hygiene, malnutrition, limited access to early stimulation and education, and inadequate caregiver support. Approximately 40% of Egyptian children under five are at risk of not reaching their developmental potential [13, 14]. National surveys reveal that only half of Egyptian parents engage in developmentally stimulating activities, with paternal involvement being notably limited [15]. Moreover, over 50% of preschool-aged children are not enrolled in early education programs, and even those with access often experience poor quality services due to overcrowding, untrained staff, and irrelevant curricula [16]. These challenges are more pronounced in rural and Upper Egypt, where poverty, illiteracy, and traditional parenting styles restrict opportunities for healthy child development [17].

Evidence shows that investment in early childhood programs yields substantial returns for individuals, families, and society. Early childhood stimulation (ECS) interventions have demonstrated positive effects on children’s cognitive and socioemotional skills, with long-term impacts on human capital development [9]. A meta-analysis of 17 randomized evaluations across 11 countries confirms that ECS programs targeting children aged 0–3, through caregiver training and engagement, improve their ability to think, communicate, and relate socially [18]. Despite the growing recognition of early childhood development (ECD) challenges in Egypt, few rigorously evaluated intervention models have been implemented that are tailored to the needs of rural families [19, 20], or grounded in internationally recognized frameworks such as the Multiple Indicator Cluster Surveys (MICS) [21, 22]. Furthermore, there is limited empirical evidence from Egypt linking caregiver stimulation practices to measurable improvements in developmental outcomes among young children, particularly in underserved areas [22–25]. In alignment with Egypt’s commitment to achieving the Sustainable Development Goals, the current study implemented a community-based intervention to enhance awareness and practices surrounding early childhood stimulation in a rural Egyptian district. This manuscript aimed first to evaluate the effectiveness of the intervention in fostering a stimulating and learning environment, using validated indicators from the Multiple Indicator Cluster Surveys. The second objective was to assess the developmental progress of participating children across key domains.

## Methodology

### Study design and setting

This manuscript forms part of a larger research initiative titled “A Community-Based Nutritional and Behavioral Intervention for Healthy Generations: Toward an Exemplary Model Village.” The overarching goal of the project is to catalyze a community-wide behavioral shift by empowering parents and caregivers of children from conception to 12 years through integrated nutritional and cognitive stimulation activities. The initiative was designed to generate sustainable impact at both individual and community levels by fostering a social movement around early child development and health promotion. Previous publications stemming from this project have focused on intervention outcomes among infants and toddlers [27]– [28].

This quasi-experimental longitudinal study with a post-test-only control group was conducted over four years, from January 2019 to January 2022, in Gharbia Governorate, Egypt. Two demographically similar villages were selected from El Mahallah El Kubra district: El Othmanyia served as the intervention site and Nemra el Basal as the matched control. Both villages share comparable sociodemographic characteristics but belong to different local administrative units to avoid contamination.

### Target group and eligibility criteria

Eligible participants were mothers of children aged six years or younger residing in the intervention village. Eligibility for inclusion in the assessment and evaluation phases of the study required full engagement in the intervention. Engagement was defined as attending at least two stimulatory sessions, completing the three-month behavioral stimulation cycle targeting key caregiving practices, and participating in scheduled quarterly follow-up visits. Only mothers who completed the entire intervention protocol were considered for outcome evaluation.

Mothers who were reached but did not fulfill these engagement criteria; specifically, 109 out of 577 were excluded from the evaluation phase. This reflects an 18.9% attrition rate.

Although this level of attrition could raise concerns of potential selection bias, we conducted a comparative analysis (S-Table 1) of sociodemographic characteristics between the excluded and included mothers. The two groups did not differ significantly across key variables, including maternal age, parental education and occupation, and child sex (*p* > 0.05 for all), thereby supporting the internal validity and representativeness of the final evaluation sample. The initial baseline data of the excluded cases were discarded to avoid bias due to incomplete exposure.

Exclusion criteria also included any mother who withdrew consent, failed to attend scheduled sessions or follow-ups. Any condition likely to confound developmental outcomes (chromosomal anomalies, severe prematurity, perinatal complications malnurtied children) were excluded, consistent with previous national screening protocols in Egypt [29]. This exclusion aligns with evidence on the national prevalence and profiles of disability types among Egyptian children, highlighting the necessity for early intervention to address preventable developmental delays [25]. These children were referred for clinical management but excluded from outcome analyses to maintain the study’s internal validity.

### Sample size and sampling technique

Based on formulas by Fleiss et al. (2003), Chow et al., and Blackwelder (1998) [30–34]. A sample size of 230 was determined to detect a 25% difference in behavioral change with 83% power and α = 0.05. Of the 577 mothers reached in the intervention village, 468 completed the full intervention. A systematic random sampling technique was applied, starting with a randomly selected mother and continuing with every other eligible mother until the target of 230 was reached. Out of 468 of the engaged mothers in the intervention (completed the intervention activities), the data of 230 mothers were targeted for assessment and evaluation.

### Phases of the study

The study progressed through three distinct phases.

**Phase I: Formative Assessment** involved baseline evaluation of the caregiving environment using a structured questionnaire to determine gaps and prioritize behavioral objectives.

**Phase II: Intervention Phase** involved a comprehensive, multi-tiered approach designed to enhance early childhood stimulation and development through caregiver engagement and community mobilization. Intervention models addressing nutrition and stimulation have been shown to enhance child growth and school achievement in the Egyptian context [33]. Moreover, Socio-emotional outcomes in Egyptian infants have been strongly associated with early caregiving environments and stimulation practices [34].

The interventions began with a six-month capacity-building program delivered by specialists from the National Research Centre of Egypt. This training was held at the rural health unit of the intervention village and was directed toward seven healthcare professionals (five nurses and two physicians), who were subsequently designated as community educators. These educators were responsible for disseminating key developmental messages to caregivers using a tailored version of the UNICEF/WHO educational toolkit. The toolkit was adapted based on the findings of the formative assessment phase to align with the local context and cultural norms. Additionally, seven community health workers (CHWs) received focused training to facilitate the recruitment, mobilization, and sustained engagement of eligible mothers in the intervention activities.

The core of the intervention targeted caregivers (primarily mothers), but also encouraging father involvement through structured health education, counselling, and training sessions. The aim was to foster three core parenting behaviors associated with enriched home stimulation:

- Allocating regular daily time for interactive play and communication with children.
- Reading or browsing picture books with children.
- Creating homemade toys from recycled materials to encourage creativity and motor skills.

Additional messages included the importance of recognizing developmental milestones and red flags for delays, promoting early numeracy and literacy activities, and reducing reliance on violent disciplinary practices in Favor of positive parenting approaches.

Each caregiver underwent a three-month behavioral change cycle, during which the three targeted behaviors were introduced sequentially, one per month until all were covered. Weekly sessions supported these changes, reinforced through home visits and community events. This process was repeated every three months with a new group of participants, resulting in 16 full implementation cycles over four years.

**Fig. 1:**
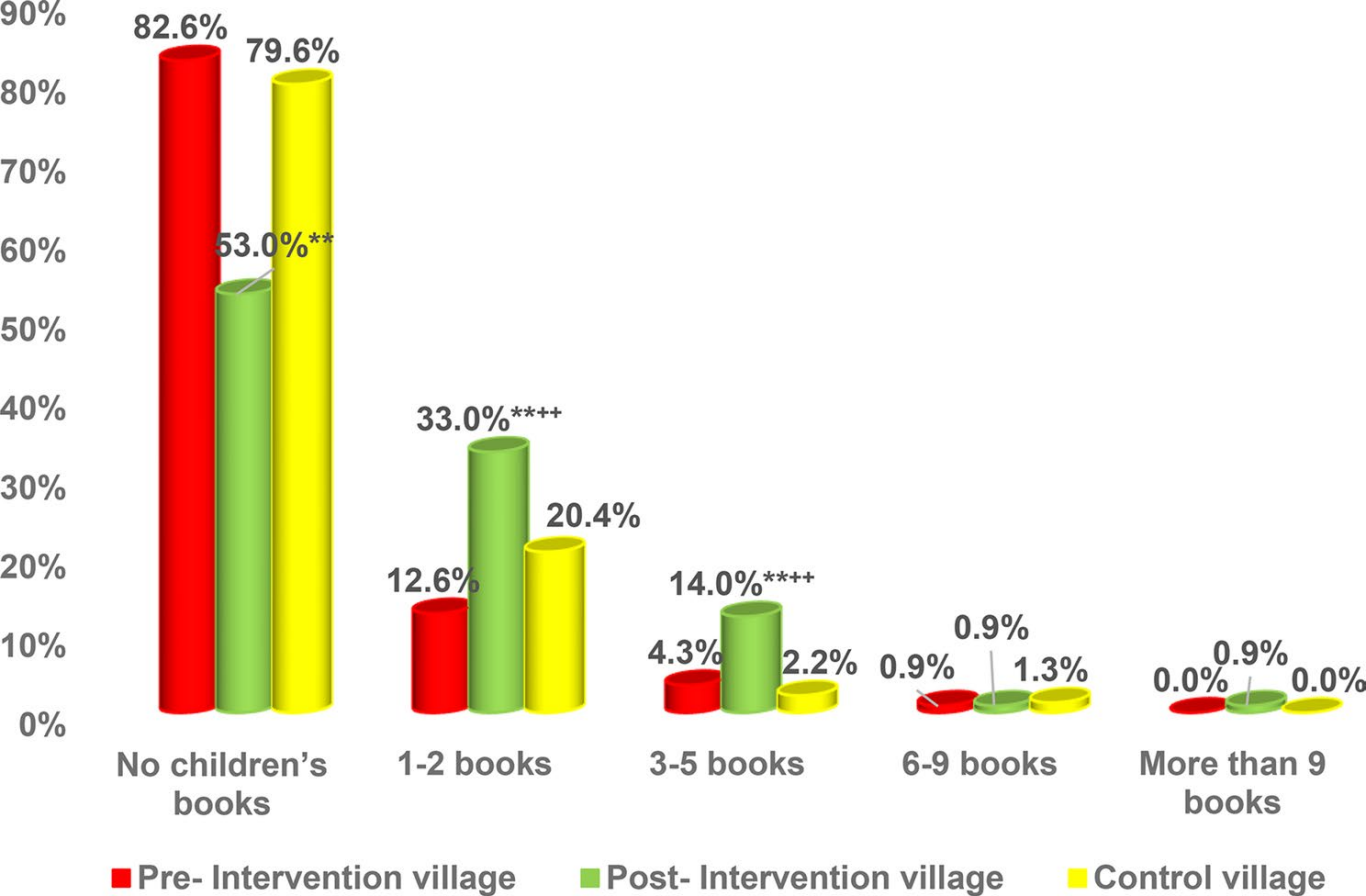
Distribution of children’s book ownership in pre- and post-intervention village and control village. Test of significance: Z test between proportion. **Highly significant at *p* < 0.01 between pre- and post-intervention village. ^++^Highly significant at *p* < 0.01 between post-intervention village and control village

The flow of intervention activities, including objectives, key messages, behavior change practices, engagement strategies, educational tools, and giveaway materials is detailed in S-Figure 1.

**Phase III: evaluation phase;** Two complementary methodological approaches were employed during the evaluation phase. First, a between -group comparison was conducted by evaluating caregiving practices in the intervention village against those in the control (non-intervention) village, which had not received any component of the intervention. This allowed for an assessment of the intervention’s relative effectiveness under real-world conditions. Developmental assessments were not conducted in the control village due to ethical and logistical considerations; specifically, screening without an established referral or intervention framework was deemed inappropriate in communities that were not receiving any developmental support, in order to avoid raising unmet expectations.

Second, a within-group comparison was carried out by assessing developmental outcomes in the same cohort of children from the intervention village before and after the intervention. This pre–post assessment design enabled the study group to serve as its own control, strengthening the internal validity of the findings.

The final evaluation took place after the full implementation of the intervention using a participatory approach. Community health workers (CHWs), under the close supervision of the study team, administered the structured and pre-validated questionnaires. These tools were used consistently at baseline and post-intervention to measure changes in both caregiver practices and child developmental outcomes.

### Key core performance indicators (KPIs)

The primary outcomes used to evaluate the success of the intervention were defined by a set of KPIs known as the FCI. These indicators were derived from the Multiple Indicator Cluster Survey (MICS), a global household survey initiative developed by UNICEF to provide internationally comparable, statistically sound estimates on key indicators related to child and maternal well-being across sectors such as health, education, and protection *(UNICEF MICS)*.

The FCI tool, developed by expert groups under UNICEF guidance and piloted across diverse settings (e.g., Brazil, Burkina Faso, Nepal, Uganda, and Zanzibar), assesses the extent to which the home environment supports early childhood development [35]. In the current study, the internal consistency of the adapted FCI domains was examined using Cronbach’s α, which demonstrated satisfactory reliability across domains: Availability of Children’s Books (α = 0.82), Variety of Play Materials (α = 0.76), Sources of Play Materials (α = 0.79), Adult–Child Interactive Activities (α = 0.84), Preschool Attendance (α = 0.88), and Paternal Involvement (α = 0.89). S-Table 2 shows the internal consistency reliability (Cronbach’s Alpha) of FCIs and subdomains in the pilot and main samples. Overall composite reliability for the total FCI scale was α = 0.86, indicating strong internal consistency with previous validation work in low- and middle-income contexts [35, 36].

Community-based models are particularly relevant in Egypt, where national screenings have identified significant developmental delays among preschool and school-aged children and stressed the need for early life interventions [14, 29]. In this study, the FCIs were adapted to local context and grouped into the following domains, aligned with international frameworks [37]:

1. Availability of Children’s Books.
  KPI: Percentage of children under the age of six who have access to three or more children’s books.

2. Variety of Play Materials.
  Toys categorized into seven functional types: musical instruments, writing/drawing items, picture books, construction materials (e.g., blocks), mobility toys (e.g., balls), educational toys (e.g., for colors/shapes), and role-playing toys (e.g., dolls, tea sets).
  KPI: Percentage of children with access to two or more types of playthings.

3. Sources of Play Materials.
  Categories include: household objects, natural/external items, store-bought toys, and homemade toys.
  KPI: Percentage of children with two or more distinct sources of play materials.

4. Adult-Child Interactive Activities.
  Activities in the preceding three days include: reading or looking at books, storytelling, singing, outdoor exploration, toy play, and naming/counting/drawing exercises.
  KPI: Percentage of families engaging in at least four of these activities.

5. Preschool Attendance.
  Percentage of children regularly attending preschool or early childhood education programs (defined as > 3 h/day for ≥ 4 days/week).

6. Paternal Involvement in Childcare.
  Proportion of fathers who actively participate in caregiving and child development activities.

All indicators were scored binarily: “Yes” = 1, “No” = 0.

### Target setting and Post-Intervention actions

Based on baseline assessment results, achievable and context-specific targets were established as follows [28]:

- For indicators initially ≤ 25%, the target was to reach ≥ 50%.
- For indicators between 26 and 50%, the target was ≥ 75%.
- For indicators between 51 and 75%, the target was 70–80%.
- For indicators already > 75%, the goal was to sustain or further improve the level.

As an ethical measure and to promote equity, all families in the control village received the same educational toolkit and promotional materials distributed in the intervention village, after the final evaluation was completed (refer to S-Figure 1).

### Data collection tools

A structured, interviewer-administered questionnaire was utilized to collect comprehensive sociodemographic data from the target groups. The collected variables included child age and sex, number of household members, parental education and occupation, ensuring alignment in socioeconomic profiles across participants. A detailed family interview component was conducted to explore potential risk factors associated with developmental delays or early childhood morbidity. These included obstetric and neonatal variables such as mode of delivery, gestational age, birth weight, feeding practices, vaccination status, birth order, maternal parity, maternal education and age, birth spacing, and broader socioeconomic conditions.

#### Direct observation

Direct observation of the child and primary caregiver was a critical component of both assessment and evaluation. Observational protocols included assessment of the child’s functional abilities, the caregiver’s responsiveness, and the overall quality of the caregiver-child interaction. In addition, physical and neurological examinations, along with vision and hearing screenings, were performed to detect any observable developmental or sensory impairments.

Developmental evaluations were tailored to each child’s age and developmental stage, ensuring relevance and accuracy in outcome interpretation. Cross-cultural discrepancies in normative child development have been documented in Egypt, underscoring the importance of local adaptation of assessment tools [38].

**Developmental Screening**: Developmental screening was conducted using the Denver II Developmental Screening Test, a validated tool designed to identify possible developmental delays in children from birth through six years of age [39]. This screening assesses four developmental domains: personal-social, fine motor-adaptive, language, and gross motor skills. The test, which includes both direct child assessment and parental reporting, was administered by trained community health workers (CHWs) and typically required 10–20 min to complete.

Children scoring below age-appropriate expectations were categorized as either “suspect” or “delayed.” Those classified as suspect underwent re-screening two weeks later to confirm findings. Children identified as delayed were referred for comprehensive evaluation by developmental and behavioral specialists. The tool has a reported sensitivity of 0.83 and specificity of 0.43 [40]. Previous national-level autism risk screenings in Egypt have demonstrated the feasibility of wide-scale developmental screening tools in primary care and community settings [41].

### Implementation to ensure validity and reliability

To maintain consistency and data quality, community educators and CHWs underwent six months of training based on adapted UNICEF/WHO materials. Intervention sessions followed a structured schedule, with supervision provided by researchers from the National Research Centre. Fidelity of message delivery and participant adherence were monitored continuously. All data collectors were blinded to study hypotheses during evaluation. Reliability analyses further supported measurement robustness: inter-rater reliability of the Denver II Screening Test, conducted on a 10% subsample (*n* = 46), yielded Cohen’s κ = 0.87 (*p* < 0.001). S-Table 2 shows domain-specific internal consistency where α = 0.81 (for fine motor), 0.84 (for language), 0.86 (for gross motor), and 0.88 (for personal-social). These coefficients are comparable with published values [39, 40], and confirm the adequacy of tool performance in the Egyptian cultural context.

### Study dependent variables

Primary dependent variables included improvements in FCI domains and developmental screening outcomes measured by the Denver II tool. Each FCI domain and developmental outcome was treated as a binary dependent variable for logistic regression analyses (coded 1 = achieved or normal, 0 = not achieved or suspected delay), enabling estimation of adjusted odds ratios for the association between intervention exposure and positive outcomes after controlling for sociodemographic characteristics.

Secondary outcomes encompassed the degree of maternal engagement, adoption of targeted caregiving behaviors, and paternal involvement in early child development, which were analyzed descriptively and compared between groups to reflect behavioral uptake and family participation in nurturing care practices.

### Statistical methods

Following data cleaning, all completed questionnaires were entered into the Statistical Package for the Social Sciences (SPSS), version 24, for analysis. Descriptive statistics were used to summarize baseline and outcome data, with quantitative variables expressed as means and standard deviations and qualitative variables presented as frequencies and percentages.

To evaluate the effect of the intervention, both within-group (pre–post) and between-group (intervention vs. control) comparisons were conducted. Pearson’s Chi-square (χ²) and Z-tests were applied to detect differences in proportions, and paired *t*-tests were used to compare continuous pre- and post-intervention means. Statistical significance was set at *p* ≤ 0.05 throughout the analysis [42].

Multivariable binary logistic regression analyses were subsequently performed to identify independent predictors of improved Family Care Indicators (FCIs) and developmental outcomes while adjusting for relevant sociodemographic factors. Each regression model included one FCI or developmental domain as the dependent variable (coded 1 = achieved/normal, 0 = not achieved/suspected delay), with participation in the community-based intervention as the main predictor (coded 1 = intervention, 0 = control). Covariates included maternal and paternal education, maternal and paternal occupation, parity and child sex.

Regression results were expressed as Adjusted Odds Ratios (AORs) with corresponding 95% Confidence Intervals (CIs). Model adequacy was evaluated using the Hosmer–Lemeshow goodness-of-fit test (*p* > 0.05 indicating acceptable fit) and Nagelkerke R² values. Absence of multicollinearity was verified using Variance Inflation Factor (VIF < 2), and all models employed the Enter method for variable entry.

This analytic approach ensured clarity and statistical rigor, providing both unadjusted and adjusted evaluations of the association between the intervention and family-care as well as developmental outcomes in the study population.

## Results

### Sociodemographic characteristics

The baseline socioeconomic characteristics were comparable between the intervention and control villages, ensuring minimal confounding by sociodemographic variables. The two villages were well-matched with no significant differences in sociodemographic characteristics. Mothers’ ages ranged from 20 to 40 years, with a mean age of approximately 28.1 years. Around 71.8% of mothers and 61.7% of fathers in both villages had attained intermediate education. A smaller proportion (10.4% to 15.7%) of mothers and 9.6 to 10.9% of fathers had university degrees. The vast majority of mothers were housewives (95.7%–96.5%), and most fathers were employed. The gender distribution of children was nearly equal across both groups (Table 1).

**Table 1:** Characteristics of the studied population of the intervention and control villages

Parameter	Intervention village (*n* = 230) *N* (%)	Control village (*n* = 230) *N* (%)	*P* value
**Gender**: Boys Girls	109 (47.4) 121 (52.6)	105 (45.7) 125 (54.3)	0.71
**Mother’s age (years)**			
Range	20–40	20–40	
Mean ± SD	28.1 ± 5.3	26.9 ± 6.1	0.98
**Maternal education**: Illiterate / primary school Prep/high school University	41 (17.8) 165 (71.8) 24 (10.4)	39 (17) 155 (69.6) 36 (15.7)	0.251
**Paternal education**: Read and write/ primary school Prep./ High school University	66 (28.7) 142 (61.7) 22 (9.6)	62 (27.0) 143 (62.1) 25 (10.9)	0.852
**Paternal employment status**: Employed (farmers/ employees/ drivers/ labor workers) Non-Employed	212 (92.2) 18 (7.8)	220 (95.7) 10 (4.3)	0.119
**Maternal employment status**: Non-working (housewives) Working	220 (95.7) 10 (4.3)	222 (96.5) 8 (3.5)	0.631

Tests of significance: X^2^ test between groups, t- test between two means, Z-test between proportions

### Changes in family care indicators Post-Intervention

Table 2 presents the post-intervention changes in seven key FCIs, comparing results between the intervention and control villages, while also examining changes from pre- to post-intervention within the intervention group. The indicators reflect core parenting behaviors targeted by the program, including early learning support, play environments, and caregiver engagement.

Post-intervention analysis revealed statistically significant improvements (*p* < 0.001) in 6 out of 7 indicators in the intervention village, both compared to their own pre-intervention status and to the matched control village. These included increased access to children’s books, availability and diversity of play materials (especially homemade toys), regular engagement in multiple learning activities, book reading practices, and preschool programs attendance. The only indicator that did not significantly change was fathers’ involvement in childcare, which remained high and stable across both time points and villages.

### Achievement of behavioral targets

The intervention successfully met or approached key behavioral targets. Achievement of targeted behavioral change goals ranged from 56.8% to 100% across indicators. For example, the proportion of children regularly attending preschool increased from 17.0% to 45.7% (100% target achievement, meeting the target of 40%). Similarly, the percentage of caregivers who reported reading picture books with their children increased markedly from 22.2% to 45.7%, reaching approximately 85% of the targeted goal. In terms of access to play resources, the proportion of children with access to two or more sources of play materials grew from 58.7% to 83%, reflecting 76% of the target achievement. Furthermore, access to three or more children’s books improved from 4.3% pre-intervention to 14% post-intervention, accomplishing 56% of the intended target **(**Table 2**).**

**Table 2:** Post-Intervention impact of the family care stimulating environment program on family care indicators among targeted groups in intervention vs. Control villages

Indicators	Intervention village				Control village *n* = 230 *N* (%)
	Pre- Intervention *n* = 230 *N* (%)	Targeted behavior Objectives as a result of the intervention	Post- Intervention *n* = 230 *N* (%)	% Target Achieved	
**Availability of children books**:					
**1- Proportion of children under 6 years of age who have 3 or more children’s books**	10 (4.3)	**To** reach 25%	32 (14.0)	56.8% of the target	5 (2.2)
P value of Z test				< 0.001+**	0.19§
P value of Z test (Post Intervention vs. Control					< 0.001**
**Availability of playthings**:					
**2- Proportion of infants and preschool children who have 2 or more of playthings**	179 (77.8)	**To be sustained**	206 (89.6)	Achieved	180 (78.3)
P value of Z test				< 0.001+**	0.92§
P value of Z test (Post Intervention vs. Control					< 0.001**
**Diversity of sources of playing materials**					
**3- Proportion of infants and preschool children who have at least 2 or more sources of playing materials of which homemade toy is a source**	135 (58.7) with 19.4% home-made toys	**Increase the proportion of children having homemade toys to 50%**	191 (83.0) with 39.6% home-made toys	76% of the target	142 (61.7) with 17.4% home-made toys
P value of Z test				< 0.001+**	0.50§
P value of Z test (Post Intervention vs. Control					< 0.001**
**Support for learning: Proportion of children who are engaged with their caregivers in developmental learning activities**					
**4- The percent of families that were keen to specify regular times for interaction with their children for at least 4 activities in the last three days**	169 (73.5)	**To be sustained**	213 (92.6)	Achieved	164 (71.3)
P value of Z test				< 0.001+**	0.60§
P value of Z test (Post Intervention vs. Control					< 0.001**
**5- The percent of families that were keen to read books in the last three days**	51 (22.2)	**to** reach 50%	105 (45.7)	85% of the target	58 (25.2)
P value of Z test				< 0.001+**	0.44§
P value of Z test (Post Intervention vs. Control					< 0.001**
**Regular attendance of preschool education program**					
**6- Proportion of children who regularly attend preschool education program > 3days/week and for 3 h or more in each day**	39 (17.0)	**to** reach 40%	105 (45.7)	100% of the target	43 (18.7)
P value of Z test				< 0.001+**	0.62§
P value of Z test (Post Intervention vs. Control					< 0.001**
**Father’s participation in child care**					
**7- Proportion of fathers involved in child care (playing)**	159 (69.1)	**To be sustained**	157 (68.3)	The supportive role of father has been maintained in families	161 (70.0)
P value of Z test				0.84+	0.84§
P value of Z test (Post Intervention vs. Control					0.69

Test of significance: Z-test between proportions

**Highly significant at *p* < 0.01, += Pre vs. Post Intervention, §=Pre intervention vs. control

### Improvement in book availability

Prior to the intervention, there were insignificant differences in children’s book ownership between the intervention and control groups. However, following the intervention, notable improvements were observed in the intervention group. The proportion of families owning 1–2 children’s books increased from 12.6% to 33.0%, which was significantly higher than the 20.4% reported in the control group (*p* < 0.01). Similarly, the percentage of families possessing 3–5 books rose from 4.3% to 14.0%, compared to only 2.2% in the control group (*p* < 0.01) (Fig. 1).

### Sources and variety of play materials

**Fig. 2:**
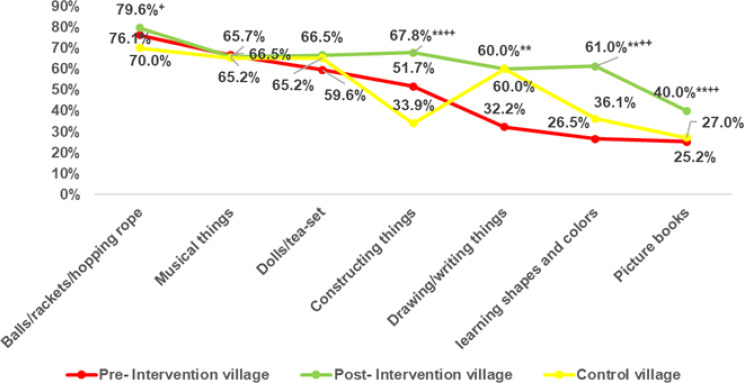
Variety of Play Materials in pre- and post-intervention village and control village. Test of significance: Z test between proportion. **Highly significant at *p* < 0.01 between pre- and post-intervention village. ^+^Significant at *p* < 0.05, ^++^Highly significant at *p* < 0.01 between post-intervention village and control village

Figure 2 illustrates the impact of the intervention on the availability of diverse play materials among children. Following the intervention, the proportion of children in the intervention group with toys designed for learning colors and shapes rose markedly from 26.5% to 61%, compared to 36.1% in the control group with a highly significant improvement (*p* < 0.01).

**Fig. 3:**
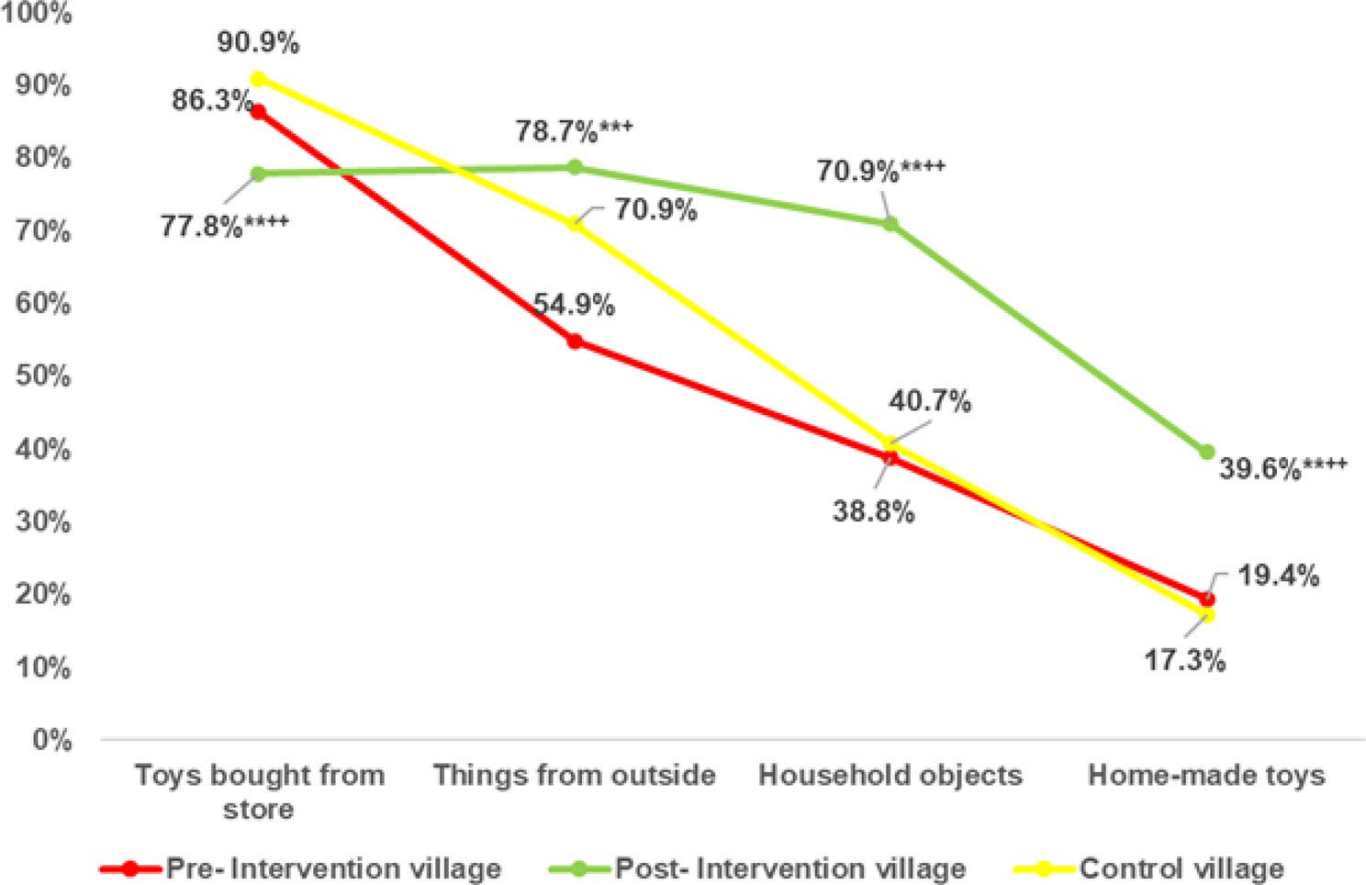
Changes in types and sources of play materials as a result of intervention versus control village. Test of significance: Z test between proportion. **Highly significant at *p* < 0.01 between pre- and post-intervention village. ^+^Significant at *p* < 0.05, ^++^Highly significant at *p* < 0.01 between post-intervention village and control village

Insignificant statistical differences were observed between the intervention village at the baseline and the control village regarding sources of play materials (e.g., store-bought toys, items from outside the home, household objects, and homemade toys). However, post-intervention results revealed significant improvements within the intervention village. The percentage of children with household objects as play materials increased significantly to 70.9%, compared to 38.8% pre-intervention and 40.7% in the control village. Moreover, access to homemade toys rose to 39.6% post-intervention from 19.4% at baseline and 17.3% in the control group (Fig. 3**).**

### Caregiver–Child communicative activities

**Fig. 4:**
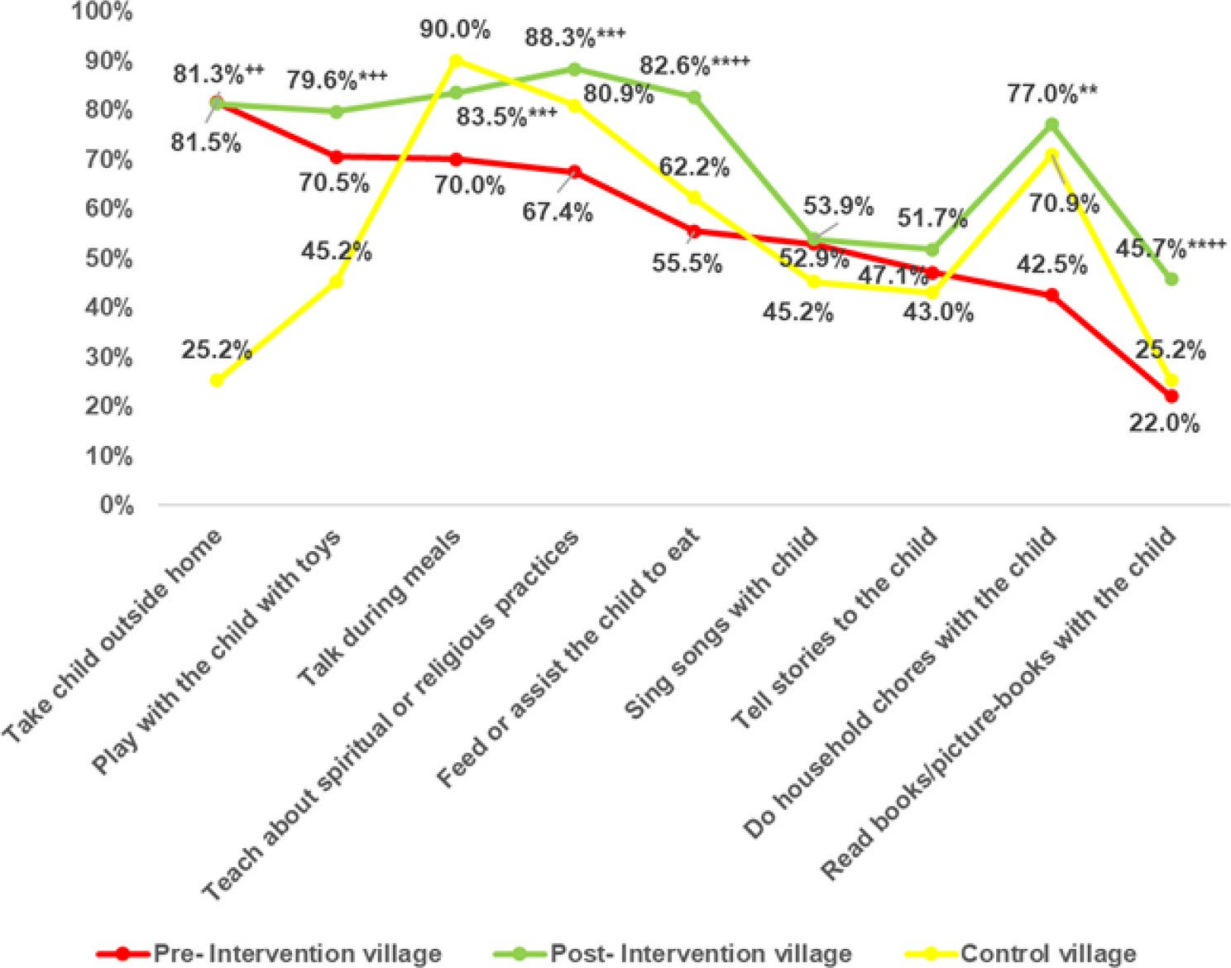
Parent-Child Interactive Activities in Intervention and Control Villages. Test of significance: Z test between proportion. *Significant at *p* < 0.05, **Highly significant at *p* < 0.01 between pre- and post-intervention village. ^+^Significant at *p* < 0.05, ^++^Highly significant at *p* < 0.01 between post-intervention village and control village

Before the intervention, there were insignificant statistical differences between the intervention and the control groups regarding most of the parent-child communicative activities. After the intervention, significant improvements were observed in several areas within the intervention group. The percentage of parents reading books or picture books with their children rose to 45.7% post-intervention, compared to 22% pre-intervention and 25.2% in the control group. Additionally, the proportion of parents assisting their children with feeding increased to 82.6%, from 55.5% pre-intervention and 62.2% in the control group (Fig. 4**).**

### Paternal involvement in childcare

**Fig. 5:**
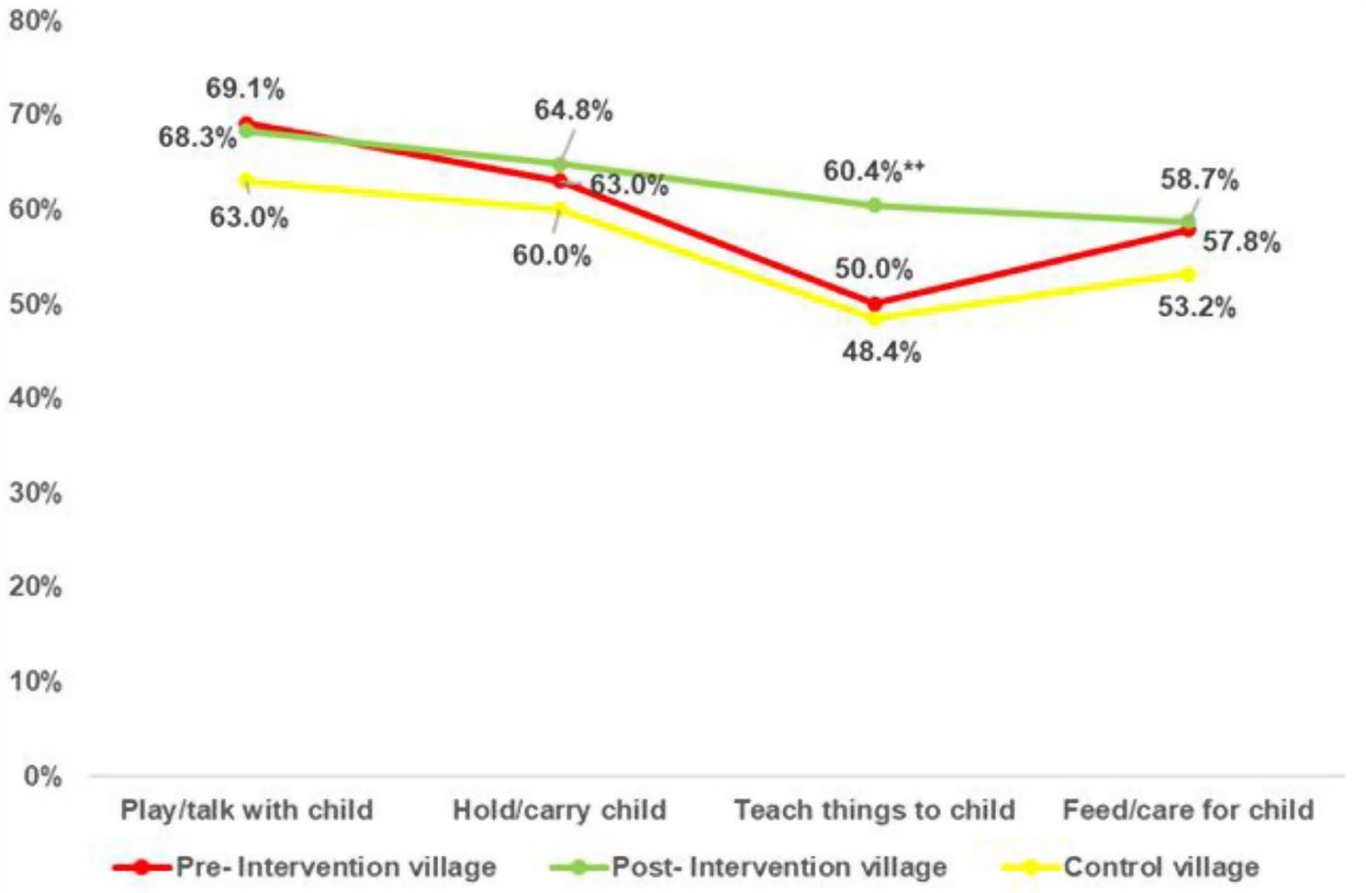
Father’s engagement in teaching and childcare activities in the intervention and control villages. Test of significance: Z test between proportion. *Significant at < 0.05 between pre- and post-intervention village. ^+^Significant at < 0.05 between post-intervention village and control village

The impact of the intervention on paternal involvement in early child care activities is shown in Fig. 5. Pre-intervention differences were not significant from that of the control village. Following the intervention, significant improvements were recorded in activities such as teaching children, which rose to 60.4% in the intervention group compared to 50% pre-intervention and 48.4% in the control group, with a statistically significant difference (*p* < 0.05).

### Developmental screening outcomes (DDST Domains)

The family care stimulation program led to a considerable impact on child development, as measured by the Denver II Developmental Screening Test. The proportion of children categorized as “normal” improved significantly across all four developmental domains. The intervention effectively enhanced developmental performance across all domains, particularly in fine motor and language skills. The fine-motor domain exhibited the most marked improvement, with normal scores increasing from 53.0% to 77.8%. The language development domain followed, improving from 59.1% to 76.1%. Gross motor scores rose from 62.2% to 83.0%, while personal-social development improved from 65.2% to 83.9%. All changes were statistically significant (*p* < 0.01) (Table 3).

**Table 3:** Pre- and post-intervention developmental status across DDST domains assessed by Denver-II screening test

DDST Domains	Pre-intervention *n* = 230 *N* (%)	Post-intervention *n* = 230 *N* (%)	*P* value
**Fine motor development** Normal scores Suspected delay	122 (53.0%) 108 (47.0%)	179 (77.8%) 51 (22.2%)	< 0.001**
**Language development** Normal scores Suspected delay	136 (59.1%) 94 (40.9%)	175 (76.1%) 55 (23.9%)	< 0.001**
**Gross motor development** Normal scores Suspected delay	143 (62.2%) 87 (37.8%)	191 (83.0%) 39 (17.0%)	< 0.001**
**Personal-social development** Normal scores Suspected delay	150 (65.2%) 80 (34.8%)	193 (83.9%) 37 (16.1%)	< 0.001**

Test of significance: Z test between proportion, **Highly significant at *p* < 0.01 between pre- and post-intervention village

Normal: No delay and a maximum of one caution, Suspect: Two or more cautions and /or one or more delays

### Adjusted associations between intervention exposure and FCIs

Table 4 shows that after adjusting for maternal age, education, occupation, parity and child sex, participation in the community-based intervention was significantly associated with improved outcomes across all FCIs domains and developmental measures. Families in the intervention village had 3.45 times more odds to own at least three children’s books (AOR = 3.41; 95% CI 1.87–6.24; *p* < 0.001) and 2.71 times more odds to have access to at least two different sources of play materials (AOR = 2.71; 95% CI 1.68–4.38; *p* < 0.001). Caregivers were also substantially more engaged in child-focused stimulation activities: those in the intervention arm had 3.22 times higher odds of engaging in four or more interactive behaviors such as reading, storytelling, and playing with their child (AOR = 3.22; 95% CI 1.97–5.25; *p* < 0.001). Shared reading practices were significantly more frequent (AOR = 2.58; 95% CI 1.54–4.32; *p* < 0.001), and regular preschool attendance (> 3 days per week) had nearly four times more odds among intervention children (AOR = 3.76; 95% CI 2.01–7.02; *p* < 0.001).

**Table 4:** Multivariable logistic regression of family care indicators adjusted for maternal and paternal characteristics

Parameters	Availability of ≥ 3 children’s books		Access to ≥ 2 sources of play materials		Access to ≥ 2 sources of homemade toys		Engagement in ≥ 4 caregiver–child activities (past 3 days)		Shared reading with child (past 3 days)		Regular preschool attendance (> 3 days/week)	
	AOR	CI	AOR	CI	AOR	CI	AOR	CI	AOR	CI	AOR	CI
**Intervention participation** (Yes is the base = 1)	3.41**	1.87	2.71**	1.68	2.84**	1.69	3.22**	1.97	2.58**	1.54	3.76**	2.01
		6.24		4.38		4.79		5.25		4.32		7.02
**Child sex** (male is the base = 1)	0.88	0.58	1.07	0.68	1.11	0.61	0.94	0.61	1.04	0.67	1.09	0.71
		1.33		1.67		2.23		1.44		1.61		1.69
**Maternal age** (years, continuous)	1.02	0.98	1.00	0.96	1.31	0.77	1.01	0.97	1.02	0.98	1.00	0.96
		1.07		1.04		2.25		1.06		1.07		1.05
**Parity (number of live births)**	0.95	0.80	0.92	0.77	0.93	0.78	0.94	0.78	0.91	0.77	0.94	0.78
		1.12		1.09		1.11		1.12		1.08		1.12
**Maternal education** (≥ intermediate is the base = 1)	1.34	0.82	1.29	0.77	1.24	0.69	1.33	0.80	1.27	0.76	1.35	0.81
		2.19		2.17		2.23		2.21		2.11		2.26
**Paternal education** (≥ intermediate is the base = 1)	1.29	0.77	1.21	0.69	1.23	0.70	1.18	0.68	1.16	0.66	1.22	0.69
		2.16		2.10		2.15		2.04		2.04		2.16
**Maternal occupation** (employed is the base = 1)	0.93	0.50	0.96	0.50	0.96	0.48	0.91	0.48	0.98	0.52	0.93	0.49
		1.72		1.83		1.70		1.74		1.83		1.77
**Paternal occupation** (employed is the base = 1)	1.02	0.98	1.15	0.60	1.17	0.61	1.10	0.57	1.09	0.56	1.14	0.58
		1.07		2.18		2.24		2.12		2.12		2.22

Variable(s) entered in each model: intervention participation, child sex, maternal age, maternal education, paternal education, maternal occupation, paternal occupation, and parity. AOR: Adjusted Odds Ratio, CI: Confidence Interval, **highly significant at *p* < 0.01. Regression type: Binary logistic regression with logit link (Enter method). Model fit diagnostics: Hosmer–Lemeshow χ² = 6.21, *p* = 0.51; Nagelkerke R² = 0.21–0.28; no multicollinearity detected (VIF < 2)

### Adjusted associations between intervention exposure and developmental outcomes

For developmental outcomes measured by the Denver II Screening Tool, adjusted models showed consistently strong associations between intervention exposure and the absence of suspected delays (Table 5). Children in the intervention group had 3.18 times more odds to have normal fine-motor development (95% CI 1.92–5.26; *p* < 0.001), 2.64 times more odds to have age-appropriate language skills (95% CI 1.63–4.27; *p* < 0.001), and 2.93 times more likely to exhibit normal gross-motor abilities (95% CI 1.75–4.91; *p* < 0.001). The strongest association emerged for personal–social development, where children from intervention households carried 3.83 times more odds to demonstrate age-appropriate social behaviors (95% CI 2.07–7.09; *p* < 0.001). Across all models, the Hosmer–Lemeshow goodness-of-fit tests were non-significant (*p* > 0.05), indicating adequate model calibration, and Nagelkerke R² values ranged between 0.21 and 0.34, reflecting moderate explanatory power typical for behavioral and developmental outcomes. No multicollinearity was detected (Variance Inflation Factor < 2 for all predictors).

**Table 5:** Multivariable logistic regression of developmental outcomes (Denver II) adjusted for maternal and paternal characteristics

Parameters	Fine motor (normal)		Language (normal)		Gross motor (normal)		Personal–social (normal)	
	AOR	CI	AOR	CI	AOR	CI	AOR	CI
**Intervention participation** (Yes is the base = 1)	3.18**	1.92	2.64**	1.63	2.93**	1.75	3.83**	2.07
		5.26		4.27		4.91		7.09
**Child sex** (male is the base = 1)	0.89	0.59	1.08	0.69	0.95	0.62	1.02	0.65
		1.33		1.69		1.46		1.61
**Maternal age** (years, continuous)	1.01	0.97	1.02	0.98	1.01	0.97	1.03	0.98
		1.05		1.07		1.05		1.09
**Parity (number of live births)**	0.97	0.82	0.93	0.78	0.92	0.77	0.94	0.77
		1.14		1.11		1.10		1.15
**Maternal education** (≥ intermediate is the base = 1)	1.23	0.77	1.30	0.79	1.20	0.74	1.28	0.74
		1.97		2.12		1.96		2.19
**Paternal education** (≥ intermediate is the base = 1)	1.18	0.70	1.20	0.70	1.17	0.69	1.22	0.69
		1.98		2.05		1.99		2.14
**Maternal occupation** (employed is the base = 1)	0.94	0.51	0.98	0.53	0.88	0.47	0.92	0.47
		1.72		1.79		1.63		1.78
**Paternal occupation** (employed is the base = 1)	1.10	0.61	1.09	0.58	1.08	0.57	1.12	0.57
		1.98		2.03		2.04		2.22

Variable(s) entered in each model: intervention participation, child sex, maternal age, maternal education, paternal education, maternal occupation, paternal occupation, and parity. AOR: Adjusted Odds Ratio, CI: Confidence Interval, **highly significant at *p* < 0.01. Regression type: Binary logistic regression with logit link (Enter method). Model fit diagnostics: Hosmer–Lemeshow χ² = 7.02, *p* = 0.46; Nagelkerke R² = 0.27–0.34; no multicollinearity detected (VIF < 2)

## Discussion

### Nurturing care and the Egyptian context

Nurturing care is a cornerstone for early childhood development (ECD), depending largely on caregivers’ knowledge and ability to create a stimulating home environment [8, 43]. In Egypt, national population-based surveys show that 8.1% of children aged 1–6 years have at least one disability, and 6.4% have developmental delays [14, 19]. Additional national studies have linked early developmental delays to nutritional, social, and perinatal risk factors [44, 45]. With 57% of Egyptians living in rural areas [46], rural communities offer vital opportunities for targeted ECD interventions.

This quasi-experimental study evaluated a structured, community-based intervention rooted in the WHO/UNICEF Care for Child Development framework. The program significantly improved multiple child developmental outcomes in a rural Egyptian setting, particularly among families with low maternal education and limited awareness of stimulation practices. Key gains were documented in caregiver-child interaction, early learning activities, access to learning materials, and developmental improvement.

### Impact on caregiver behavior

The intervention strengthened maternal engagement through educational content, hands-on skill-building, and peer support. These sessions enhanced understanding of early childhood’s critical role in brain and socioemotional development [9, 47]. Mothers gained awareness of developmental milestones and milestone cards enabled early identification of developmental concerns [10, 48]. Mothers showed improved responsiveness to children’s cues, and adopted positive practices such as reading, storytelling, and interactive play. Such outcomes align with prior studies affirming the role of parental education in supporting child development [26, 48]. Using a comparable approach, behavioral improvements mirrored the outcomes observed in Egyptian health education programs for anemia and diabetes [49] and among diabetic patients [50].

### Changes in the home environment

Post-intervention, households had greater access to diverse play and reading materials, including cost-effective, homemade items. This resource shift stimulated creativity and sensory learning, supporting the literature on environmental enrichment and child development [51].

### Parents’ communication and teaching activities

Caregiver-child communication improved substantially after the intervention. The proportion of mothers engaging in shared book reading rose from 22% to 46%, and access to learning toys increased to 61%. Mothers also engaged more frequently in teaching activities such as naming, singing, and storytelling, all of which enhance language development and emotional bonding [52, 53].

### Child developmental outcomes

Participation in the intervention was associated with statistically significant gains across all assessed developmental domains. After adjustment for potential confounders, children in the intervention group remained significantly more likely to have improved home stimulation environments and normal developmental screening outcomes across all domains. These findings reinforce the robustness of the observed effects while acknowledging the study’s non-randomized design. These outcomes correspond with previous studies linking caregiver-led stimulation and structured activities with early neurodevelopmental progress [53–55]. Importantly, improvements were seen in a population with high baseline vulnerability, indicating that such interventions can be transformative even in resource-constrained settings. Notably, the observed developmental improvements cannot be attributed solely to cognitive stimulation; rather, they reflect the synergistic effect of the comprehensive intervention, which integrated nutritional support and behavioral guidance. This is supported by extensive literature demonstrating that integrated early childhood development (ECD) programs—those combining health, nutrition, and psychosocial stimulation have a greater and more sustained impact on child development than single component interventions [9, 56, 57]. This also aligns with earlier Egyptian findings showing the combined role of nutrition and stimulation in improving cognitive functions among primary school children [58].

The adjusted results demonstrate a robust and coherent pattern of association between participation in the community-based program and multiple aspects of nurturing care and child development. The intervention appears to have strengthened caregiver behaviors related to early learning, responsiveness, and stimulation, consistent with evidence that home-based and community-delivered parenting programs can significantly enhance developmental outcomes in early childhood [9, 59, 60]. The largest adjusted effects were observed for personal–social development and preschool attendance, suggesting that structured group sessions and home-based guidance may have enhanced caregivers’ confidence, consistency, and social interaction with children [5, 61]. Improvements in fine- and gross-motor skills point to greater opportunities for physical play and skill practice at home [62], while the gains in language outcomes, though slightly smaller, indicate early shifts in communication-oriented activities that often require longer periods to consolidate [63, 64].

From a broader perspective, the pattern of adjusted odds ratios (ranging 2.6–3.8) across both behavioral and developmental domains underscores the intervention’s multidimensional benefits. Although the quasi-experimental, post-test-only design limits causal inference, the adjusted regression findings strengthen confidence that the observed associations are not merely attributable to underlying sociodemographic differences. They align closely with the principles of the WHO/UNICEF Nurturing Care Framework, which emphasizes responsive caregiving, early stimulation, and enriched home environments as key determinants of optimal child development [8, 10]. Collectively, these results suggest that integrating structured caregiver education and home-stimulation guidance into routine primary health care and maternal–child services may represent an effective, low-cost pathway for reducing early developmental disparities in rural Egyptian and comparable low-resource settings [26–28].

### Supportive community engagement

Community health actors played a key role in maintaining intervention momentum. Trained CHWs, nurses, and physicians reinforced content, tracked developmental progress, and minimized dropout. Even mothers with inconsistent attendance benefited from community reinforcement.

Father engagement, though modest, improved post-intervention, particularly in educational interactions. This is a noteworthy contribution as rural paternal involvement in ECD is typically low due to entrenched gender roles. Our findings provide practical examples of feasible strategies to foster modest but impactful paternal involvement in such settings, adding to prior evidence that highlights the benefits of engaged fatherhood [65].

### Community-Based early education support

The intervention was embedded into local systems through structured CHW follow-up, regular home visits, and the creation of community resources. Small-scale libraries and nursery enrolment campaigns promoted sustained early education efforts. The rise in nursery attendance from 17% to 45.7% reflects a broader community-level shift toward valuing structured early learning [66]. Behavioral change was further driven by participatory learning methods, leadership support, and integration into existing maternal and child health platforms. This helped bridge the gap between home-based learning and formal education systems [66, 67].

### Monitoring and evaluation mechanisms

The study employed systematic monitoring tools through FCIs to track caregiver-child interaction, access to learning materials, and early educational engagement. Weekly sessions, home visits, and feedback mechanisms supported continuous assessment. CHWs were equipped to detect early delays and strengthen referral pathways. This collaborative model strengthened referral systems and ensured sustainability. The successful use of FCIs confirms their cultural adaptability for culturally and economically diverse populations [28, 35].

### Strengths and limitations

This study’s principal strength lies in its community-based, quasi-experimental design, which allowed assessment of real-world behavioral and developmental outcomes in a rural, low-resource setting. By operationalizing the WHO/UNICEF Care for Early Childhood Development framework through trained community health workers (CHWs) and local healthcare staff, the intervention demonstrated how structured caregiver education can be effectively integrated within existing primary healthcare systems.

The random selection of participants, standardized training of field teams, and use of validated assessment tools enhanced both internal validity and reproducibility. Unlike clinic-based or cross-sectional studies, this design enabled longitudinal monitoring of family care practices and developmental progress under routine community conditions, reinforcing its ecological validity and policy relevance.

Nonetheless, several limitations warrant acknowledgment. First, time constraints among participating mothers occasionally limited session attendance; however, neighborhood social networks and peer diffusion helped sustain exposure to key intervention messages. Second, developmental screening was not conducted in the control village for ethical and logistical reasons, restricting baseline comparisons. Third, a measurable attrition rate was observed. Although excluded and included participants did not differ significantly across sociodemographic characteristics, potential selection bias cannot be fully excluded.

In summary, this community-based intervention—anchored in the WHO/UNICEF nurturing care framework; was associated with significant improvements in children’s fine and gross motor, language, and personal-social development, as well as in caregiver–child interaction and home learning environments. Its low-cost, scalable model highlights the feasibility of leveraging CHWs and primary care platforms to reduce early developmental disparities in rural Egyptian and comparable low-resource contexts.

## Conclusion and recommendations

This study provides credible evidence that structured, community-based interventions can be significantly associated with improved early childhood development (ECD) outcomes in low-resource rural settings. Through caregiver education and locally delivered support, participants demonstrated measurable gains in child-rearing practices and developmental indicators. Integrating such community-based models into Egypt’s existing primary health-care (PHC) and maternal–child health systems could represent a sustainable pathway to promote equitable early-childhood outcomes across underserved populations.

To ensure scalability and institutional sustainability, embedding this model within ongoing PHC initiatives; such as growth-monitoring and immunization programs would help institutionalize early-stimulation and parenting-support components as part of routine health services. Collaboration with the Ministry of Health and Population is essential to align implementation with national child-development strategies and to strengthen multisectoral coordination.

### Future directions

Although causal inference is limited by the quasi-experimental, post-test-only design, the adjusted findings highlight the potential of integrating structured caregiver-training and early-stimulation components within PHC settings. Future work should focus on scaling this approach to other rural and peri-urban areas, adapting it to diverse cultural contexts, and evaluating long-term developmental trajectories and school readiness. Establishing a formal monitoring framework; leveraging community health workers, mobile-health technologies, and routine follow-up visits—will enable continuous tracking of Family Care Indicators beyond the study period. Further cost-effectiveness analyses and strategies to enhance paternal engagement are warranted to strengthen policy relevance, equity, and social inclusivity.

## Supplementary Information

Below is the link to the electronic supplementary material.

Supplementary Material 1

Supplementary Material 2

Supplementary Material 3

## Data Availability

The datasets generated and/or analyzed during the current study are not publicly available due to ethical considerations involving human subjects and the presence of sensitive health-related information that could compromise participant privacy. Additionally, portions of the data are currently being used in ongoing analyses intended for future publication. However, data may be made available from the corresponding author upon reasonable request and subject to institutional and ethical approval.

## Abbreviations

ECD: Early childhood development
ECS: Early childhood stimulation
MICS: Multiple Indicator Cluster Surveys
CHWs: Community Health Workers
KPIs: Key Core Performance Indicators
FCIs: Family Care Indicators
MICS: Multiple Indicator Cluster Survey
DDST Domains: Developmental Screening Outcomes
