# Supplementary material for: Bridging early development gaps in rural Egypt: a community-based approach to equitable childhood care
Source: Int J Equity Health. 2025 Dec 18;25:18. doi: 10.1186/s12939-025-02728-4 (PMC12821242; doi:10.1186/s12939-025-02728-4)
Supplement: Supplementary file 1 — Supplementary Material 1 [file 12939_2025_2728_MOESM1_ESM.docx]

| **Indicators of stimulating environment** |
| --- |
| I. Availability of children’s books: represented by the percentage of children under age 6 have three or more children’s books |
| II. Variety of play materials (7 items), which categorized toys according to their purpose and included; items that produce or perform music, items for writing and drawing, Children's picture books (not school books), Items designed for building, stacking, and construction (blocks), items for mobility (bats, balls, etc.), toys that teach colors and shapes, Items for role-playing (tea set, dolls, etc.)  Availability of playthings: represented by the percentage of children who have 2 or more of playthings |
| III. Sources of play materials (4 items), which identified where the play materials came from; Household objects, Things from outside, Toys bought from store, Home-made toys  Diversity of sources of playing materials: represented by the percentage of children who have two or more sources of playing materials |
| IV. Play activities’ (6 items), which identified specific types of activities done by any adult in the home with the child in the previous three days: Read books or look at picture-books with child, tell stories to child, sing songs with child, take child outside home place, play with the child with toys, spend time with child in naming things, counting, drawing  Communicating activities are represented by the: percentage of families that are keen to specify regular times for interaction with their children in four or more activities in the last three days |
| V. The percentage of children who regularly attend preschool education program for >3hours/day for ≥4 days |
| VI. Proportion of fathers involved in child care |

**Supplementary File Figure 1**
