# Supplementary material for: Bridging early development gaps in rural Egypt: a community-based approach to equitable childhood care
Source: Int J Equity Health. 2025 Dec 18;25:18. doi: 10.1186/s12939-025-02728-4 (PMC12821242; doi:10.1186/s12939-025-02728-4)
Supplement: Supplementary file 2 — Supplementary Material 2 [file 12939_2025_2728_MOESM2_ESM.docx]

# S-Table 1: Comparison of sociodemographic characteristics between included and excluded mothers

| Characteristic | Included (n = 468) | Excluded (n = 109) | Test Statistic | p-value |
| --- | --- | --- | --- | --- |
| Maternal age (years), mean ± SD | 28.9 ± 5.7 | 29.3 ± 5.9 | t = 0.52 | 0.603 |
| Maternal education (% ≥ intermediate) | 62.0% | 59.6% | χ² = 0.22 | 0.639 |
| Paternal education (% ≥ intermediate) | 68.4% | 66.1% | χ² = 0.19 | 0.664 |
| Maternal occupation (% employed) | 18.2% | 19.3% | χ² = 0.07 | 0.795 |
| Paternal occupation (% employed) | 89.1% | 87.9% | χ² = 0.10 | 0.752 |
| Child sex (% male) | 52.6% | 54.1% | χ² = 0.07 | 0.788 |

Notes: Values are presented as mean ± standard deviation (SD) for continuous variables or percentages for categorical variables. Comparisons between groups were conducted using independent-samples t-tests for continuous variables and Pearson’s Chi-square (χ²) tests for categorical variables. All p-values > 0.05, indicating no statistically significant differences between included and excluded participants.
