# Supplementary material for: Bridging early development gaps in rural Egypt: a community-based approach to equitable childhood care
Source: Int J Equity Health. 2025 Dec 18;25:18. doi: 10.1186/s12939-025-02728-4 (PMC12821242; doi:10.1186/s12939-025-02728-4)
Supplement: Supplementary file 3 — Supplementary Material 3 [file 12939_2025_2728_MOESM3_ESM.docx]

S-Table 2: Internal consistency reliability (Cronbach’s Alpha) of family care indicators, their subcomponents and Developmental Domains

| **FCIs and sub items** | **Cronbach's Alpha*** |
| --- | --- |
| Availability of ≥ 3 children’s books | .820 |
| Availability of ≥ 2 children’s playthings | .763 |
| Availability of ≥ 2 homemade toys | .792 |
| Shared reading with child (past 3 days) | .812 |
| Engagement in ≥ 4 children’s activities (past 3 days) | .836 |
| Regular preschool attendance (past 3 days) | .884 |
| Fathers involved in child care (playing) | .886 |
| Musical things | .821 |
| Drawing/writing things | .807 |
| Picture books | .816 |
| Constructing things | .825 |
| Balls/rackets/hopping rope | .808 |
| Learning shapes/colors | .812 |
| Dolls/tea-set | .815 |
| Household objects | .823 |
| Things from outside | .820 |
| Toys from stores | .815 |
| Homemade toys | .826 |
| Read books/picture-books with the child | .812 |
| Tell stories to the child | .812 |
| Sing songs with child | .816 |
| Take child outside home | .813 |
| Play with child with toys | .817 |
| Do household chores with child | .822 |
| Teach about spiritual or religious practices | .817 |
| Feed or assist child to eat | .815 |
| Talk during meals | .821 |
| Father play and talk with child | .829 |
| Father feed/take care of child | .823 |
| Father hold/carry child | .825 |
| Father teaches things to child | .821 |
| Fine motor delay | .812 |
| Language delay | .839 |
| Gross motor delay | .856 |
| Personal–social delay | .884 |

- * The study-reported reliability for 10% of sample (pilot study) for FCI: cronbach's alpha= 0.863. Denver II The study-reported reliability for 10% of sample (pilot study) for Developmental Domains: cronbach's alpha= 0.869. (*p* < 0.001).
